# Elimination of *Schistosoma mansoni* Adult Worms by Rhesus Macaques: Basis for a Therapeutic Vaccine?

**DOI:** 10.1371/journal.pntd.0000290

**Published:** 2008-09-17

**Authors:** R. Alan Wilson, Jan A. M. Langermans, Govert J. van Dam, Richard A. Vervenne, Stephanie L. Hall, William C. Borges, Gary P. Dillon, Alan W. Thomas, Patricia S. Coulson

**Affiliations:** 1 Department of Biology, University of York, York, United Kingdom; 2 Department of Parasitology, Biomedical Primate Research Centre, Rijswijk, The Netherlands; 3 Department of Parasitology, Leiden University Medical Centre, Leiden, The Netherlands; George Washington University, United States of America

## Abstract

**Background:**

Among animal models of schistosomiasis, the rhesus macaque is unique in that an infection establishes but egg excretion rapidly diminishes, potentially due to loss of adult worms from the portal system via shunts or death by immune attack.

**Principal Findings:**

To investigate this, six rhesus macaques were exposed to *Schistosoma mansoni* cercariae and the infection monitored until portal perfusion at 18 weeks. Despite a wide variation in worm numbers recovered, fecal egg output and circulating antigen levels indicated that a substantial population had established in all animals. Half the macaques had portal hypertension but only one had portacaval shunts, ruling out translocation to the lungs as the reason for loss of adult burden. Many worms had a shrunken and pallid appearance, with degenerative changes in intestines and reproductive organs. Tegument, gut epithelia and muscles appeared cytologically intact but the parenchyma was virtually devoid of content. An early and intense IgG production correlated with low worm burden at perfusion, and blood-feeding worms cultured in the presence of serum from these animals had stunted growth. Using immunoproteomics, gut digestive enzymes, tegument surface hydrolases and antioxidant enzymes were identified as targets of IgG in the high responder animals.

**Significance:**

It appears that worms starve to death after cessation of blood feeding, as a result of antibody-mediated processes. We suggest that proteins in the three categories above, formulated to trigger the appropriate mechanisms operating in rhesus macaques, would have both prophylactic and therapeutic potential as a human vaccine.

## Author Summary

Infection with blood-dwelling schistosome worms is a major cause of human disease in many tropical countries. Despite intensive efforts a vaccine has proved elusive, not least because the chronic nature of the infection provides few pointers for vaccine development. The rhesus macaque appears unique among animal models in that adult worms establish but are eventually lost. We investigated whether this was due to pathological or immunological causes by monitoring the fate of a schistosome infection, and were able to rule out escape of worms from the portal system as a result of egg-induced vascular shunts. A substantial worm population established in all animals but there was a wide variation in the numbers recovered at 18 weeks. We observed a strong inverse association between the rapidity and intensity of the IgG response and worm burden. Rather than an acute lethal attack, immune-mediated elimination of worms appeared to be a prolonged process directed against vital components of exposed surfaces, causing worms to starve to death. We suggest that if the mechanisms deployed by the rhesus macaque could be replicated in humans by administration of key recombinant antigens, they would form the basis for a vaccine with both prophylactic and therapeutic properties.

## Introduction

Schistosomiasis remains a major public health problem in the Tropics, with tens of millions infected and many more at risk [1]. It has been estimated that greater than 250,000 deaths per annum are directly attributable to the disease [2], and the subtle morbidities associated with chronic infection have a more serious impact than hitherto credited [3]. Treatment relies on a single drug (praziquantel) to eliminate the adult worms but, as this has no prophylactic properties and is ineffective against larval schistosomes [4], a vaccine would augment efforts to control and ultimately eradicate the disease. Once established in the human portal tract adult *Schistosoma mansoni* are long-lived [5], revealing their ability to deploy effective immune evasion strategies. In pre-pubertal children there is little evidence for immune-mediated prevention of worm recruitment, as a result of which the prevalence and intensity of infection rise gradually with age [6]. Even in those adults who are apparently resistant to reinfection, suggesting the development of acquired immunity, no mechanisms have been defined on which a vaccine might be based [7].

The difficulties inherent in research on human schistosomiasis have entailed the use of laboratory animal models, with some early studies being undertaken in the rhesus macaque (*Macaca mulatta*) [8]–[10]. In this species, exposure to a moderate number of cercariae elicited protection against a challenge given four to five months later while the adult worms that engendered the immune response were apparently unaffected [9]. By analogy with tumor transplantation, the term concomitant immunity was proposed as an explanation [11]. Resistance to challenge was also demonstrated in mice with a chronic *S. mansoni* infection [12] but was subsequently shown to be an artefact of pathology, not immune-mediated killing [13]. The porta-caval shunts that developed in mice as a result of egg-induced hepatic pathology prevented challenge larvae from establishing by providing them with an escape route from the portal to the pulmonary vasculature, and even permitted adult worms from the primary infection to exit and pass to the lungs [13].

A salient feature of the rhesus macaque host is that an infection becomes patent but, above a threshold worm burden, egg output declines over the ensuing weeks to months [10],[14]. As in the mouse, such a decline might be explained by the escape of worms through developing porta-caval shunts, meaning that they could no longer deposit eggs in the intestinal wall. Conversely, if shunts do not develop in the rhesus macaque, the observed decline in egg output with time could reflect immune elimination of the primary worm burden. We sought to establish which of these two contending hypotheses provided the most likely explanation. A clear demonstration of anti-adult worm immunity would open a new route towards the elusive goal of a schistosome vaccine, potentially one with therapeutic properties.

## Methods

### Parasite exposure and sampling regime

The study used six adult female rhesus macaques (mean age 15.8±7.1 years, mean weight 5.0±1.2 kg) from the colony at the Biomedical Primate Research Centre (BPRC), Rijswijk, The Netherlands. The experimental protocol was approved by the Institutional Animal Care and Use Committee at BPRC and the Biology Department Ethics Committee, University of York. Animals were exposed to 1000 *S. mansoni* cercariae (Puerto Rican isolate; Department of Parasitology, Leiden University Medical Centre, The Netherlands) via the shaved abdominal skin for 30 minutes, under tiletamine-zolazepam (Zoletil; Virbac, Barneveld, NL) anaesthesia supplemented with ketamine hydrochloride (Alfasan International, Woerden, NL). Serum was obtained from finger prick bleeds at 2-wk intervals between weeks 6 and 14, and by intravenous sampling prior to infection and at perfusion (wk 18).

### Indirect estimates of infection intensity

Fecal samples were collected overnight at 1 or 2 wk intervals from wk 6. Eggs per gram of feces was determined from three individual samples/animal/time point, using the Percoll technique [15]. Soluble circulating anodic antigen (CAA), released into the bloodstream from the parasite's gut, was detected by ELISA using specific monoclonal antibodies [16].

### Measurement of portal pressure and porta-caval shunting

Animals were anaesthetised as above and laparotomized to measure portal blood pressure via the superior mesenteric vein, followed by the injection of 15×10^6^ carbonised inert microspheres (15 µm diameter) to measure the extent of porta caval shunting, both as previously described for rodents [17]. Animals were maintained under deep anaesthesia for 15 minutes before perfusion, after which the lungs and liver were weighed and samples retained. The fractional distribution of microspheres in lungs and liver+perfusate was then estimated by counting aliquots of tissues digested overnight in 5% KOH at 60°C [18].

### Recovery of worms from the portal vasculature

Rhesus macaques received an intravenous injection of heparin followed by an overdose of anaesthetic before ligaturing the aorta and vena cava to isolate the portal vasculature. Portal perfusion was performed as described for the olive baboon [19], and the worms were fixed in formal saline after counting under a stereomicroscope. Reference worms were recovered from C57BL/6 mice (University of York Animal Facility) seven weeks after exposure to 200 cercariae, and fixed in formal saline.

### Confocal and electron microscopy

Worms were prepared for confocal microscopy by post-fixation in AFA (alcohol/40% formaldehyde/glacial acetic acid, in the ratio 85∶10∶5), staining for 30 min in Langeron's Carmine [20], differentiation in 70% acid alcohol, clearing, and mounting in DPX (VWR International Ltd). Optical slices were obtained with a Bio-Rad MRC-1000 confocal microscope, with excitation at 514 nm from a 25 mW argon ion laser and a 585 nm long pass emission filter. Kalman averaging was carried out over 12 frames. For electron microscopy, worms were post-fixed in 2.5% glutaraldehyde/4% paraformaldehyde in 100 mM PBS, pH 7.2, at 4°C overnight [21].

### Effect of serum on worm growth

Mechanically transformed schistosomula [22] were cultured in M169 medium (Invitrogen, Paisley, Scotland) supplemented with 5% normal rhesus serum, 1% glutamine and 1% penicillin/streptomycin in 24-well plates, 95% O2∶5%CO2, 37°C for 4 days. Medium was then replaced and 1% rhesus erythrocytes added to cause transformation to blood-feeding liver-stage worms. On day 8, they were pooled and re-plated in 50% M169∶50% test or control serum plus 1% erythrocytes, with a medium change on day 16. Test sera were pooled from the two animals with highest and the two with lowest worm burdens at perfusion. Control serum and erythrocytes from an uninfected rhesus macaque were the gift of Covance Laboratories, Harrogate, UK. On day 18, the three worm populations were photographed on an Optiphot-2 microscope (Nikon, Kingston, UK) at ×100 with a TK-1070E camera (JVC, London, UK), and the mean cross-sectional surface area of individuals (n = 28 to 48) measured as an index of growth. Significance was determined by paired sample *t* test against the uninfected serum control.

### Antibody responses

Levels of IgM and IgG antibodies against soluble adult worm proteins (SWAP) were determined by ELISA, as described previously [23]. Wells were probed with alkaline phosphatase-conjugated rabbit anti-monkey IgG (Sigma-Aldrich, Poole, UK) or goat F(Ab')_2_ anti-human IgM (Biosource International, Nivelles, Belgium), diluted 1∶2000, colour developed using p-Nitrophenyl phosphate substrate (Sigma) and absorbance read at 405 nm. Total IgE concentrations were determined by ELISA [24] on plates coated with 1∶2000 rabbit anti-human IgE (DakoCytomation, Ely, UK) and IgE binding detected with a 1∶1000 dilution of peroxidase-conjugated rabbit anti-human IgE (DakoCytomation). A rhesus macaque serum was calibrated against a human serum immunoglobulin standard (The Binding Site, Birmingham, UK).

### Probing of gut secretions and tegument surface proteins by Western blotting

A stimulated adult worm secretory preparation (SASP), derived predominantly from the worm gut, was generated as described [25], apart from the final concentration step to <1 ml volume by centrifugation at 3000 rpm, 4°C using Vivaspin 20 µl 5 K MWCO filter units (VWR International Ltd, Lutterworth, UK); a 20 µl aliquot of protease inhibitor cocktail (Sigma) was added before storage at −20°C. A tegument surface preparation (TSP) was generated by incubating adult worms for 1 h in RPMI 1640 (Invitrogen) plus 10 mM HEPES containing phosphatidyl inositol phospholipase C (Sigma) at 1.25 units/ml. The culture supernatant was concentrated and protease inhibitor cocktail added before storage at −20°C. The protein composition of these two preparations, analysed by tandem mass spectrometry (MS), will be reported elsewhere (Hall, S.L. et al., Borges, W.C. et al., manuscripts in preparation). Concentrated supernatants were buffer-exchanged using 25 mM Tris, pH 7.5 before separation of 30 µg of protein on pre-cast mini 2D gels [26]. For TSP, 1D separations were performed on 1.0 mm 4–12% NuPAGE Bis-Tris gels (Invitrogen) [26]. ID and 2D separations were transferred to PVDF membranes [26], for probing with a 1∶500 dilution of rhesus macaque sera, before incubation with 1∶1000 alkaline phosphatase-conjugated rabbit anti-rhesus IgG antibody (Sigma), both for 1.5 h at RT, and development using 5-bromo-4-chloro-3-indolyl phosphate/nitro blue tetrazolium (Sigma). Antibody targets were identified by matching Western blots to gels separated under identical conditions, by visual inspection or Phoretics Evolution Software (Non-linear Dynamics, Newcastle, UK).

## Results

### Blood feeding and fecal egg output decline from soon after patency

**Figure 1:**
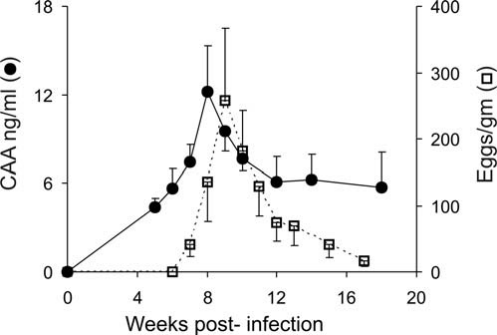
Blood feeding and fecal egg output decline from soon after patency. Worm gut function as an indicator of physiological status, determined by the concentration of CAA in the bloodstream of rhesus macaques (•). Fecundity of the worm population, revealed by fecal egg output (□). Values are mean + or − SE. n = 6 animals.

CAA and fecal egg output were monitored, as indirect indicators of the status of the schistosome infection (Figure 1). The production of CAA rose steadily as worms began blood feeding and reached maturity, with mean values peaking at week 8 before declining to a plateau from week 12 onwards. Eggs were first detected in the feces at week 7, with mean numbers peaking at week 9 before declining towards the baseline; in two cases egg excretion reached zero by the end of the study. Although data from individual animals showed a wide variation for the two indirect indicators (Table 1), their peak values revealed that appreciable numbers of parasites established in all rhesus macaques. However, there was a large discrepancy in the number of worms recovered at perfusion (range 12–708; Table 1), signifying that many had been lost from the portal system in some animals.

**Table 1:** Parasitological and associated parameters for individual rhesus macaques.

Rhesus #	Peak fecal eggs^a^ (wk)	Peak CAA^b^ (wk)	Worm burden	Portal pressure^c^	% portal shunting
R1	69±34 (8)	9.65 (9)	12	20.6 (2.02)	0
R2	292±72 (11)	15.0 (8)	708	10.3 (1.01)	0.02
R3	361±35 (10)	26.5 (8)	80	32.3 (3.17)	4.0
R4	688±164 (9)	11.36 (7)	91	22.3 (2.19)	96.0
R5	93±8 (11)	7.84 (10)	249	8.1 (0.79)	0.02
R6	476±53 (9)	8.91 (8)	31	8.9 (0.87)	2.4

a Mean±S.E. of triplicate samples.

b ng/ml.

c cm H_2_O (kPa).

### Most surviving worms are morphologically degenerate

**Figure 2:**
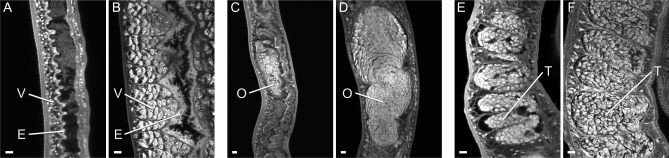
Most surviving worms are morphologically degenerate. Confocal microscopy on selected tissues of adult worms recovered from the portal system of rhesus macaques (A, C, E) and mice (B, D, F), and stained with Langeron's carmine. A) female posterior lacking vitelline lobules (V) and with greatly simplified intestinal epithelium (E) versus B) abundant vitelline lobules and normal intestinal epithelium with lamellate extensions. C) female mid body showing shrunken ovary (O) with few recognizable oocytes versus D) normal ovary with large number of healthy oocytes. E) shrunken testes (T) with lacunae and few sperm versus F) normal testes structure. All images are the same magnification. Bar = 10 µm.

The declining values of the indirect indicators might reflect worm death or a more subtle deterioration in physiological function. In fact, when worms were counted a large proportion, particularly the females, had a pallid appearance as they lacked hematin pigment in their intestines. Many females were also shrunken compared to the mature equivalent from mice, with bodies approximately two-thirds the normal width. Confocal microscopy of the pallid female worms (n = 15) revealed that the intestinal epithelium was thinner and its luminal surface lacked the numerous lamellar extensions seen in normal female worms from mice (n = 9; Figure 2A cf. B). A decline in reproductive function of the pallid females was reflected by the greatly shrunken appearance of the ovary (Figure 2C) that contained very few oocytes compared to normal worms (Figure 2D). The vitelline lobules were strikingly absent in the pallid worms (Figure 2A cf. normal worms Figure 2B), as were forming eggs in the ootype (data not shown). The degeneration of the reproductive system was not confined to females. Pallid males (n = 7) had reduced numbers of spermatocytes in their testicular lobules, giving the contents a shrunken appearance with lacunae (Figure 2E) versus the tight packing of spermatocytes and maturing spermatozoa in the normal testis (n = 4; Figure 2F). A few females of normal appearance had an ovary with healthy oocytes, a forming egg in the ootype and sperm present in the proximal part of the oviduct that acts as a receptaculum seminis; this last is indicative of functionally active male worms.

**Figure 3:**
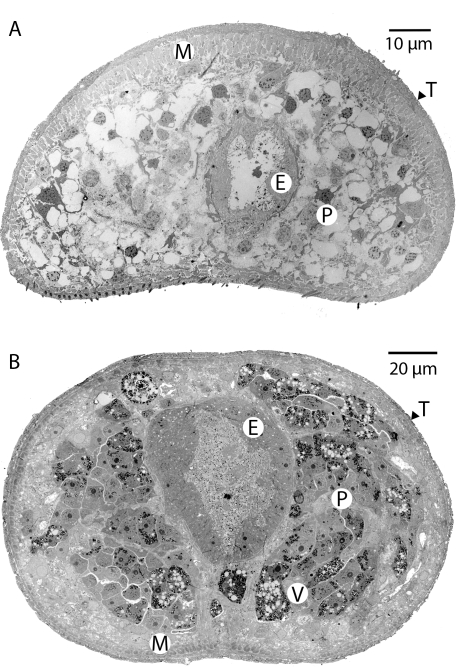
Cells internal to the body wall musculature are atrophied. Electron micrograph of a transverse section of the posterior of A) pallid female worm from rhesus macaque. The syncitial tegument (T), body wall musculature (M) and intestinal epithelium (E) are intact but cells of the parenchyma (P) and vitelline lobules (V) are devoid of content apart from prominent central nuclei. The gut lumen is virtually empty. B) posterior of female worm from mouse, with abundant vitelline lobules and gut lumen full of partly digested blood.

These degenerative changes were further highlighted by an ultrastructural comparison. The cellular organization in the posterior half of the female body, comprising numerous individual vitelline cells packed with peripheral egg-shell precursor granules in worms from mice (Figure 3B), was absent in pallid worms from rhesus macaques (Figure 3A). The parenchyma appeared almost completely devoid of content, including glycogen and lipid stores, although individual cell membranes and isolated nuclei were evident. The absence of food in the gut lumen contrasted with the abundance of SEi-digested blood in the normal worm gut (Figure 3A cf. B). Electron microscopy confirmed the degenerative state of the intestinal epithelium in pallid worms. Although plasma membranes were intact, the cytoplasm was less dense, nuclei were more rounded, and endoplasmic reticulum and Golgi apparatus were lacking, implying little or no protein synthesis (Figure S1A). In contrast, the surface syncitial tegument of the parasite had a normal pitted appearance with the usual cytoplasmic inclusions (Figure S1B). The subtegumental circular and longitudinal muscles that make up the body wall, together with the muscles surrounding the gut epithelium, also appeared relatively intact.

### Worm loss is not due to escape via portal shunts

Egg-induced changes to the portal vasculature were investigated as a potential cause of worm loss, using hepatic portal blood pressure and the extent of porta-caval shunting as indicators. Values for the former ranged from 8.1 to 32.3 cm H_2_O (Table 1), and portal shunting was negligible with the exception of one animal where 96% of the microspheres was recovered from the lungs (Table 1). There was no correlation between the worm burden, portal pressure and the extent of shunting. Collectively, these data indicate that the integrity of the portal system had not been compromised by egg deposition.

### Humoral responses are implicated in declining worm fitness and death

**Figure 4:**
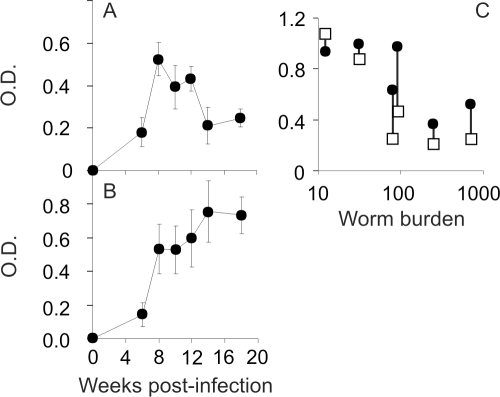
Humoral responses are implicated in declining worm fitness. Antibody responses during infection monitored by ELISA using SWAP as coating antigen. A) IgM and B) IgG. Values are mean ±SE, n = 6 animals. C) IgG levels at weeks 8 (□) and 18 (•) for individual animals plotted against the number of worms recovered at week 18.

We investigated whether the declining physiological status of worms was correlated with the humoral immune response. IgM, probed with SWAP, peaked at week 8 before gradually declining over the period of worm deterioration (Figure 4A) whereas IgG levels rose to a sustained plateau, with considerable variation between individual animals (Figure 4B). The values for IgG level at weeks 8 and 18, plotted against final worm burden, fell into 3 groups (Figure 4C). In the two animals with lowest worm recoveries, levels were already high at week 8 and remained so at week 18. Conversely, in the two with highest burdens, levels were low at week 8 with only small increments apparent by week 18. The animals with intermediate burdens had the largest increments in IgG levels between the two sampling times. Levels of total IgE showed only a small perturbation from normal (4–5 IU) around week 8 but had returned to background by week 12 (data not shown).

When blood-feeding worms were cultured in vitro with different serum pools to determine any deleterious effect on their physiology, a differential effect on growth but not on viability was observed over the 18-day test period. Thus, the mean cross-sectional surface area (mm^2^±SE) of worms in medium containing serum from the low burden pool (0.139±0.013) was approximately 50% less (*P*<0.001) than those supplemented with normal (0.233±0.013) and high burden (0.269±0.022) sera, which did not differ significantly from each other (n = 46, 37 and 28 worms, respectively).

### Antibodies recognize gut secretions and tegument surface proteins

**Figure 5:**
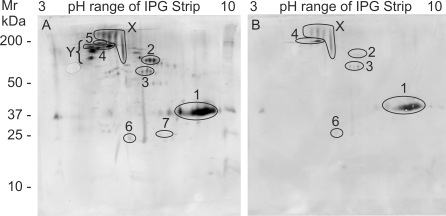
Antibodies recognize adult worm secreted proteins. Western blots of 2DE separations of SASP probed with A) high titer, low worm burden serum (R6) and B) low titer, high worm burden serum (R5). Region X is a group of highly reactive protein spots not detected by Sypro Ruby or Coomassie staining of the corresponding gel. Region Y is a group of highly reactive protein spots present only in trace amounts on the gel. SASP proteins matched to the blot are 1. asparaginyl endopeptidase, 2. unknown function, 3. thioredoxin glutathione reductase, 4. Sm200 surface protein, 5. α2 macroglobulin, 6. thioredoxin peroxidase, 7. GST 28.

To determine whether the IgG response was directed against secreted and/or accessible surface proteins we developed two corresponding novel antigen preparations. Separation of SASP by 2D electrophoresis revealed a complex mixture of proteins, many of which could be identified by tandem MS (Figure S2). When blots were probed with individual rhesus sera, only a minority of constituents was immunoreactive, with largely quantitative rather than qualitative variations observed between the six animals. The same basic pattern was present but at a higher intensity in the animals with the low burden and high titre (Figure 5A), and *vice versa* (Figure 5B). Targets identified by tandem MS on gels matched to the higher intensity blot are annotated in the figure. Two groups of highly reactive spots on the blots were either not detected on the gels by Sypro Ruby or Coomassie staining (Figure 5 Region X), strongly indicative of glycan epitopes, or were present only in trace amounts that made tandem MS identification problematic (Figure 5 Region Y). The former group is likely to comprise high molecular weight mucins while the two identities obtained for the latter (Sm200 and α2 macroglobulin) are indicative of tegument surface components.

**Figure 6:**
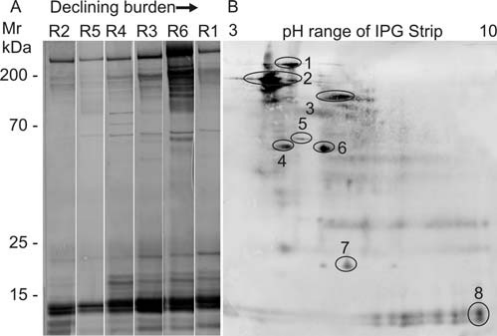
Antibodies recognize proteins exposed on the adult worm tegument. Western blots of separated TSP, probed with individual rhesus sera. A) 1DE separation showing that the complexity of serum reactivity is inversely proportional to final worm burden. B) 2DE separation probed with serum from the most reactive animal (R6). Targets identified by matching the 2D blot to an identical gel are 1. α2 macroglobulin, 2. Sm200, 3. unknown function, 4. unknown function, 5. HSP70, 6. Alkaline phosphatase, 7. Sm22.6, 8. LMWP.

A relatively simple mixture of proteins was revealed by 2D separation of TSP, some sufficiently abundant for identification by tandem MS (Figure S3). TSP is a more scarce resource than SASP so probing with individual sera was restricted to Western blots of 1D separations (Figure 6A). The complexity of serum reactivity increased in inverse proportion to the final worm burden. Both high (280 kDa) and low (14, 10, 8 kDa) molecular weight bands were strongly recognized by all animals whilst more numerous bands were evident in the four with medium to low burdens, especially R6. Sufficient material was accumulated to perform a second 2D separation, under identical conditions to the gel in Figure S3, for blotting and probing with R6 serum (Figure 6B). The principal reactive targets identified by tandem MS were Sm200, alkaline phosphatase, α2 macroglobulin, LMWP and Sm22.6.

## Discussion

In this study we have attempted to discover why the fate of a primary *S. mansoni* infection in the rhesus macaque differs from that in other permissive primate hosts [27]. A notable feature of our data was the wide range of worm recoveries at 18 weeks but the peak values for fecal egg output and level of circulating parasite antigens indicate that a substantial population established in all animals. Independent validation was provided by five adult rhesus macaques, infected under identical conditions by one of us and perfused at 8 weeks, where a mean of 43% parasite maturation was observed (range 152 to 718 worms) [28]. Perfusion data from earlier studies revealed 49% maturation at 6–7 weeks [29] and reduced primary burdens between 12 and 27 weeks relative to 8 weeks [10]. These data on actual worm numbers, together with the two indirect estimates of infection intensity, point to a gradual but appreciable loss of the established worm burden in some animals. This is contrary to the impression given by advocates of concomitant immunity that adult worms of a primary infection are unaffected by the response they provoke [11],[30].

To determine whether worms were able to escape from the portal vasculature via porta-caval shunts, as occurs in chronically infected mice [13],[17], we measured portal pressure and any associated loss of vascular integrity in the rhesus macaques. Three animals exhibited portal hypertension on the basis that normal mean values for portal pressure in this species are around 7.4 to 9.8 cm H_2_O (0.73 to 0.96 kPa) [10],[31] and values >14 cm H_2_O (1.37 kPa) are considered hypertensive [32]. These data concur with an earlier study in which a small number of rhesus macaques had portal hypertension after schistosome infection [10]. However, as we observed only a single animal with (substantial) portal shunting, translocation of worms from the portal system can be ruled out as the reason for loss of adult burden.

The absence of a pathophysiological explanation strengthens the case for an immunological mechanism. Direct evidence for inhibitory factors in the circulation is provided by the retarded growth of blood-feeding worms when cultured with a serum pool from low burden animals, compared to sera from high burden and normal animals. IgG is the most likely candidate since the mean profile of this isotype rises over the period when the indicators of worm number are declining, unlike those for IgM and total IgE that show an early rise and fall. The low levels of total IgE are unusual for infection with helminths but may reflect diminished immunostimulation. This could be due to the early cessation of egg laying, which normally drives Th2 polarization [33], and/or the reduced worms. The kinetics of IgG production is consistent with the pattern of worm elimination, with the rapid responder animals having the lowest burdens by 18 weeks and vice versa. Indeed, IgG had reached high levels in the rapid responder animals at 8 weeks, before worm elimination began, providing strong evidence that it was the cause rather than an effect of worm death. Given that the high burden animals still had low IgG levels at week 18, it appears that a threshold of specific antibody must be reached before deterioration in worm viability commences. Thereafter, we propose that sustained immunological pressure over weeks to months, not an acute lethal hit, is the cause of worm death. Support for the idea of a threshold is provided by earlier studies with animals given a low dose of cercariae where egg output continued undiminished over a prolonged period [14],[34]. Our interpretation is that the antigenic stimulus in this situation was insufficient to promote high antibody levels.

The appearance of worms at perfusion is a key pointer to the reason for their demise. The lack of blood and hematin in the gut, especially of the females that have a higher nutrient/energy requirement [35], indicates cessation of feeding that would contribute to a reduction in gut-derived circulating antigens. The capacity to acquire nutrients across the tegument surface [36] would partly compensate for loss of gut function but the vacuolated parenchyma denuded of glycogen and lipid stores reveals its inadequacy for long-term worm maintenance. The associated atrophy of vitellaria and ovaries explains the declining egg production and hence fecal egg output. Parenthetically, a reduced fecundity and shorter length of female *S. japonicum* worms, recovered from rhesus macaques at 19 versus 6 weeks, has been reported but without commentary [37]. The structural integrity of the tegument, gut epithelium and muscles we observed suggests that these tissues are crucial to survival, although it is notable that the gut epithelium showed a significant loss of biosynthetic machinery associated with blood feeding. In this context it has recently been demonstrated by RNA interference that gut Cathepsin D is essential for blood digestion and worm maturation [38]. Overall, the degenerative changes point to a process of worm starvation leading to death from organ failure. We infer from the egg output and circulating antigen data that in the rapid responder animals the process begins approximately 10 to 12 weeks after infection, with worms taking several weeks to expire.

Antigens released from or exposed by the live parasite provide a potential stimulus for the production of effector antibodies. TSP contains some of the most “external” proteins from the tegument, hence the ones likely to be accessible to antibodies while SASP contains the secreted proteins of the gut epithelium plus some tegument constituents released during short-term culture. The quantitative rather than qualitative differences in the reactivity of individual rhesus sera to TSP and SASP, suggest that the gradual recognition of specific targets coincides with an increasing ability to eliminate the worm population. We were able to identify digestive enzymes in gut secretions, tegument surface hydrolases and a number of antioxidant enzymes, plus proteins of unknown function especially at the tegument surface, as antibody targets.

We believe that the unusual ability of the rhesus macaque to eliminate adult worms is mediated by IgG but the mechanism is unclear. Our data do not support rapid opsonisation and/or complement fixation leading to lysis and/or leukocyte attack, since surface-adherent cells were not evident on starving worms. Similarly, a lack of leukocyte adherence [39] has been reported on adult worms from mice, in spite of IgM, IgG and Complement proteins at the tegument surface [40],[41]; in this host the complement cascade appears to be arrested at C4 [42]. An alternative mechanism, by analogy with well-characterized autoimmune responses, could be antibodies operating in a blocking (as in Myasthenia gravis [43]) or stimulatory (as in Grave's disease [44]) capacity. We envisage blocking antibodies impacting on nutrient uptake or the ability of worms to combat oxidative stress, while antibodies stimulating receptors could trigger hyperactivity. Due to genetic variability, some worms will be able to resist immune attack for longer than others; similarly, the speed and intensity with which a rhesus macaque responds to the schistosome infection will dictate the timing of its worm elimination.

In reappraising the rhesus macaque model we have shifted the emphasis from the more common perception of a resident worm population protecting against further parasite recruitment [11] to anti-adult immunity against a primary infection in a self-cure process [27]. This interpretation provides a novel rationale for the development of a schistosome vaccine that would be both therapeutic and prophylactic, thereby providing the ultimate control tool. Tegument surface proteins and gut secretions appear to be the source of key antigens. Indeed, two recent studies have demonstrated the protective potential in rodents of a tegument surface tetraspanin [45] and an anti-oxidant enzyme [46], the latter involving anti-adult worm immunity. The inventories of gut and tegument proteins that we have obtained by proteomic analysis [40],[41] provide the basis for a reverse vaccinology approach [47] to identify the most promising candidates.

## Supporting Information

Figure S1 Electron micrographs of epithelial surfaces of worms from rhesus macaques. A) intestinal epithelium (E) showing rounded nuclei, sparse abbreviated surface lamellae and the absence of endoplasmic reticulum and Golgi apparatus in the cytoplasm. (L) lumen, (P) parenchyma. B) tegument syncytium (T) showing normal pitted appearance and cytoplasmic inclusions. The underlying circular and longitudinal muscles (M) show the characteristic actin/myosin filament organization and mitochondria (Mt) with numerous cristae.
(11.60 MB TIF)

Figure S2 2DE gel of the soluble proteins that comprise SASP, stained with Sypro Ruby. Proteins identified by MS/MS are circled, and their corresponding details shown.
(53.33 MB TIF)

Figure S3 2DE gel of the soluble proteins that comprise TSP, released when live adult worms are incubated with PIPLC, stained with SyproRuby. Proteins identified by MS/MS are annotated directly on the gel.
(1.31 MB TIF)
