# Supplementary figures and images for: Elimination of Schistosoma mansoni Adult Worms by Rhesus Macaques: Basis for a Therapeutic Vaccine?
Source: PLoS Negl Trop Dis. 2008 Sep 17;2(9):e290. doi: 10.1371/journal.pntd.0000290 (PMC2553480; doi:10.1371/journal.pntd.0000290)

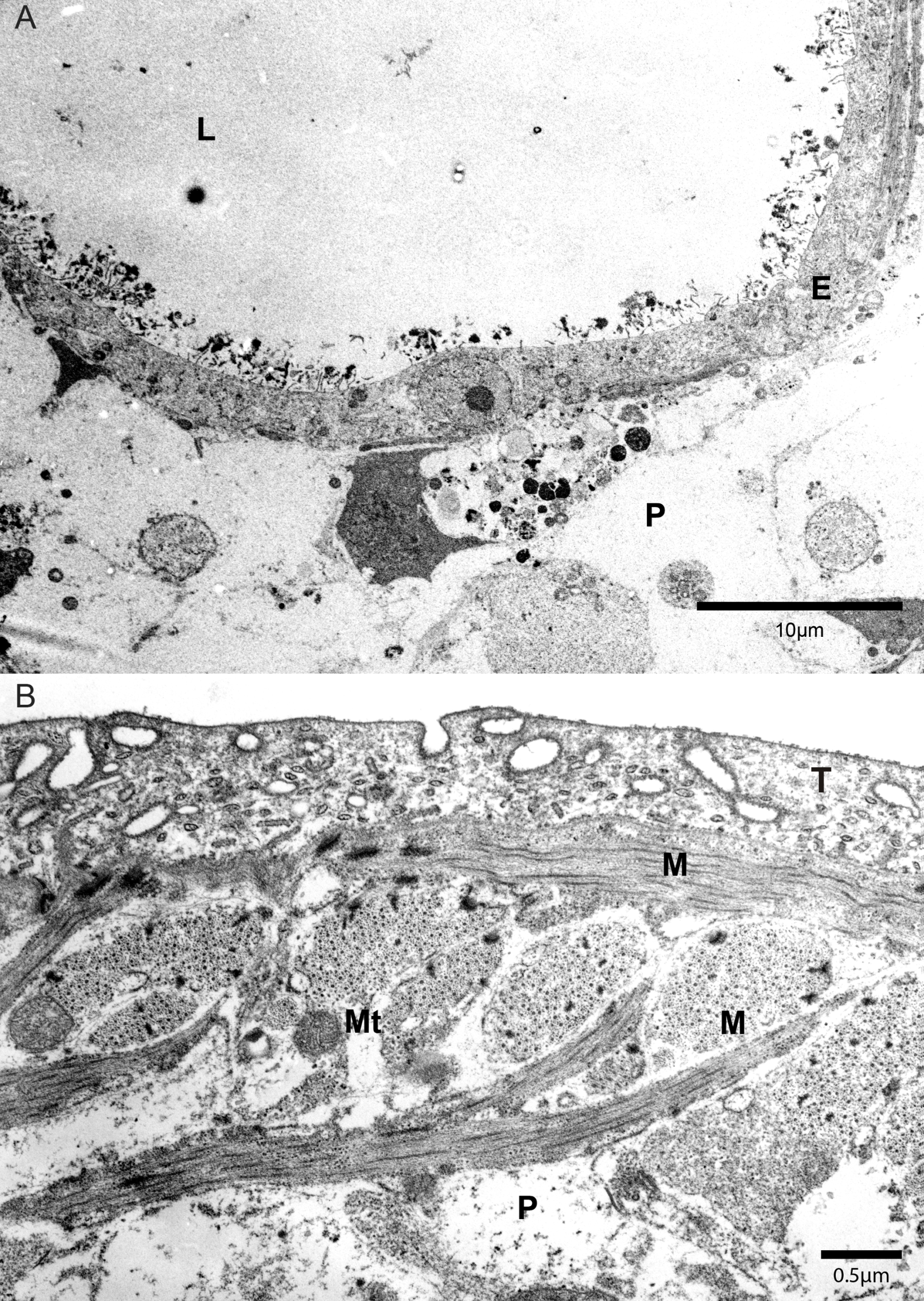

Supplement: Figure S1 — Electron micrographs of epithelial surfaces of worms from rhesus macaques. A) intestinal epithelium (E) showing rounded nuclei, sparse abbreviated surface lamellae and the absence of endoplasmic reticulum and Golgi apparatus in the cytoplasm. (L) lumen, (P) parenchyma. B) tegument syncytium (T) showing normal pitted appearance and cytoplasmic inclusions. The underlying circular and longitudinal muscles (M) show the characteristic actin/myosin filament organization and mitochondria (Mt) with numerous cristae. (11.60 MB TIF) [file pntd.0000290.s001.tif]

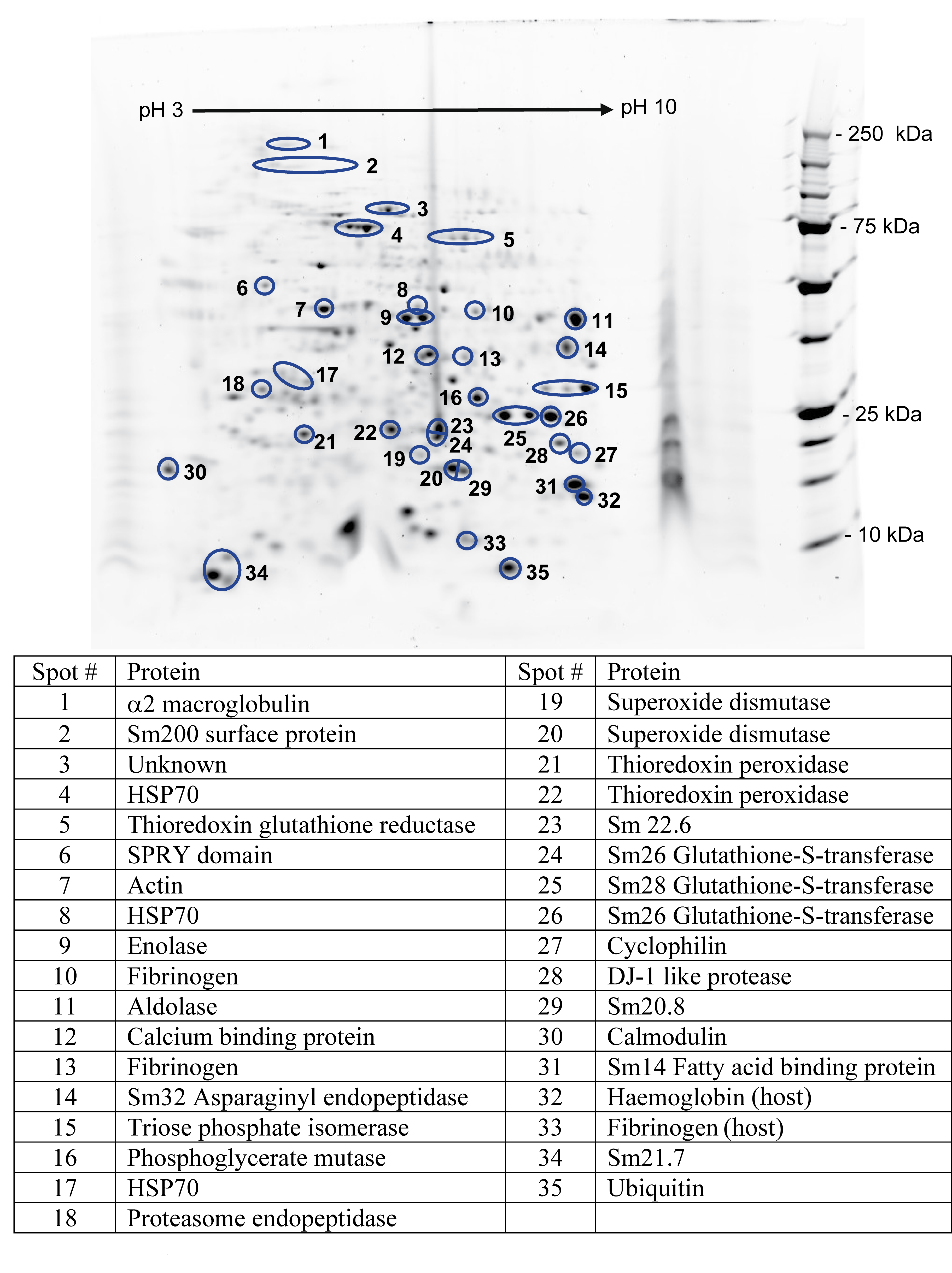

Supplement: Figure S2 — 2DE gel of the soluble proteins that comprise SASP, stained with Sypro Ruby. Proteins identified by MS/MS are circled, and their corresponding details shown. (53.33 MB TIF) [file pntd.0000290.s002.tif]

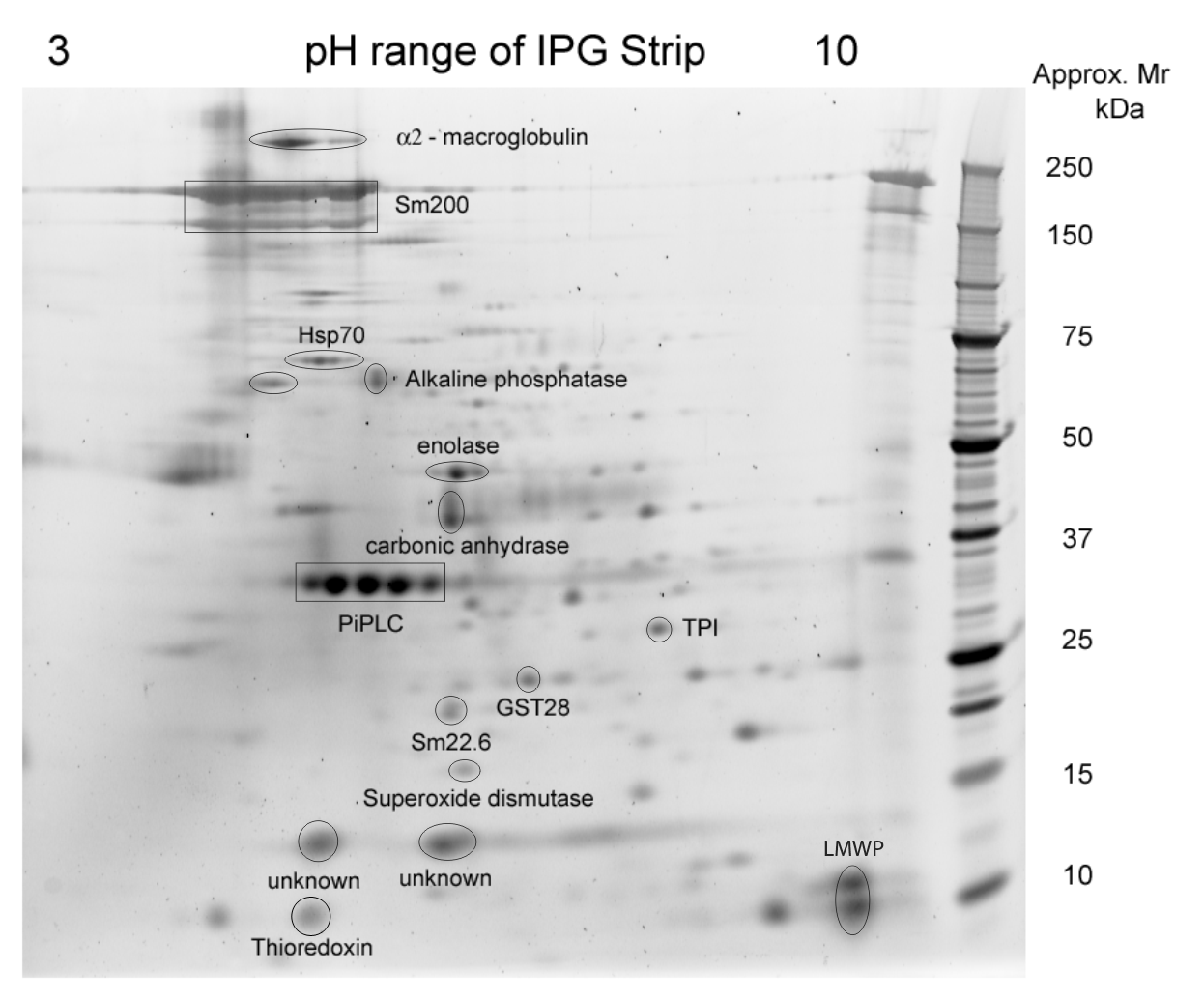

Supplement: Figure S3 — 2DE gel of the soluble proteins that comprise TSP, released when live adult worms are incubated with PIPLC, stained with SyproRuby. Proteins identified by MS/MS are annotated directly on the gel. (1.31 MB TIF) [file pntd.0000290.s003.tif]
